# Complex microstructures of ABC triblock copolymer thin films directed by polymer brushes based on self-consistent field theory

**DOI:** 10.1186/1556-276X-9-359

**Published:** 2014-07-17

**Authors:** Zhibin Jiang, Chang Xu, Yu dong Qiu, Xiaoliang Wang, Dongshan Zhou, Gi Xue

**Affiliations:** 1 Department of Polymer Science and Engineering, Key Laboratory of High Performance Polymer Materials and Technology of Ministry of Education, State Key Laboratory of Coordination Chemistry, Nanjing National Laboratory of Microstructures, School of Chemistry and Chemical Engineering, Nanjing University, Nanjing 210093, China

**Keywords:** ABC triblock copolymer, Polymer brush, Morphology, Thin film, Self-consistent field theory, Phase diagram

## Abstract

The morphology and the phase diagram of ABC triblock copolymer thin film directed by polymer brushes are investigated by the self-consistent field theory in three dimensions. The polymer brushes coated on the substrate can be used as a good soft template to tailor the morphology of the block copolymer thin films compared with those on the hard substrates. The polymer brush is identical with the middle block B. By continuously changing the composition of the block copolymer, the phase diagrams are constructed for three cases with the fixed film thickness and the brush density: identical interaction parameters, frustrated and non-frustrated cases. Some ordered complex morphologies are observed: parallel lamellar phase with hexagonally packed pores at surfaces (LAM_3_^*ll*^-HFs), perpendicular lamellar phase with cylinders at the interface (LAM^⊥^-CI), and perpendicular hexagonally packed cylinders phase with rings at the interface (C_2_^⊥^-RI). A desired direction (perpendicular or parallel to the coated surfaces) of lamellar phases or cylindrical phases can be obtained by varying the composition and the interactions between different blocks. The phase diagram of ABC triblock copolymer thin film wetted between the polymer brush-coated surfaces is very useful in designing the directed pattern of ABC triblock copolymer thin film.

## Background

Block copolymers consisting of chemically distinct polymers linked by a covalent bond at one end have the ability to self-assemble into a variety of ordered nanostructures such as lamellae (LAM), hexagonally packed cylinders (HEX), and body-centered cubic (BCC) spheres and more complex structures such as gyroid (G) in melts and solutions [1-7]. This unique characteristic of block copolymers provides possibilities for their potential applications in nanoscience, such as molecular template and nanotubes. Therefore, block copolymers have attracted a great deal of attention both in theory and experiment.

Self-assembly and phase separation in diblock copolymers have been well studied both theoretically and experimentally in the last few decades [8-14]. The phase behavior of diblock copolymers melts confined in a parallel slit or in a thin film has been extensively studied [15-25]. When the number of distinct blocks increases from two, i.e., ABC triblock copolymer, the complexity and variety of self-assembled structures are increased dramatically [1,26-39]. If a surface or interface exists, the microdomain morphologies and the kinetics of microdomain ordering can change significantly. The complex and rich phase behaviors depend not only on molecular parameters, such as the interaction energies between distinct blocks and the architectures of block copolymers, but also on external variables, such as electric fields [40,41], chemically patterned substrates [42-50], and interfacial interactions [4,51-54]. The ABC linear triblock copolymer thin films confined between two hard walls have been intensively investigated theoretically [55-58]. Feng and Ruckenstein [59] studied ABC melts in thin films by Monte Carlo simulations and showed that the microdomain morphology can be very complicated and is affected by the composition, the interactions, and even the geometry of the confinement. Ludwigs et al. [60] observed a highly ordered hexagonally perforated lamella structure based on an ABC triblock copolymer thin film.

The previous work mainly concentrated on phases of several compositions of ABC triblock copolymer by varying the film thickness or the interfacial interaction. As we know, the polymer brush-coated surface is good from the energy view [30,31]. It is equivalent to changing the surface-polymer interaction as polymer brush acts as a soft surface [30,31,61,62]. Experimentally, random copolymers were used to control the wetting behavior of block copolymer [63,64]. The results showed that the ordered structures can be easily obtained by changing the property of the surfaces or substrate, i.e., the interaction between the polymer and the surfaces. Ren et al. [61,62] observed the structure transformation of the AB diblock copolymer thin film by tailoring the grafting density of the coated surface or the concentration of the copolymer. In order to know the whole phase behavior of ABC triblock copolymer thin film confined between two parallel polymer brush-coated surfaces, we use a combinatorial screening method based on the real space implementation of the self-consistent field theory (SCFT), originally proposed by Drolet and Fredrickson for block copolymer melts [65,66,57,58] to search the equilibrium microphases of ABC linear triblock copolymers confined between the two parallel polymer brush-coated hard surfaces in three dimensions. In the present work, we concentrate on the thin film regime with film thickness of several *R*_g0_. By continuously varying the compositions of the block copolymer, the morphologies are obtained, and the phase diagrams are constructed for three different cases of interaction parameters: (1) identical interactions between three different components, (2) frustrated condition, and (3) non-frustrated condition.

## Methods

We assume the ABC triblock copolymer melt is confined between two parallel polymer brush-coated hard surfaces with a distance *L*_*z*_ along the *z*-axis. There are *n*_c_ ABC triblock copolymers with polymerization degree *N* and *n*_g_ polymer with polymerization degree *P* (here, we take *P* = *N*) grafting on the two parallel surfaces. Each copolymer chain consists of *N* segments with compositions (average volume fractions) *f*_A_ and *f*_B_ (*f*_C_ = 1 – *f*_A_ – *f*_B_), respectively. The ABC triblock copolymer and the grafted polymers (brush) are assumed to be flexible, and the mixture is incompressible with each polymer segment having a statistical length *a* and occupying a fixed volume $\rho_{0}^{-1}$. The two parallel surfaces coated by the polymer brush are horizontally placed on the *xy*-plane at *z* = 0 and *L*_z_ + *a*, respectively. The volume of the system is *V = L*_*x*_*L*_*y*_*L*_z_, where *L*_*x*_ and *L*_*y*_ are the lateral lengths of the surfaces along the *xy*-plane and *L*_z_ is the film thickness. The grafting density is defined as *σ* = *n*_g_*a*^2^/(2*L*_*x*_*L*_*y*_). The average volume fractions of the grafted chains and copolymers are *φ*_g_ = *n*_g_*N*/*ρ*_0_*V* and *φ*_c_ = *n*_c_*N*/*ρ*_0_*V*, respectively.

In the SCFT, one considers the statistics of a single copolymer chain in a set of effective external fields *w*_*i*_, where *i* represents block species A, B, and C or grafted polymers. These external fields, which represent the actual interactions between different components, are conjugated to the segment density fields, *ϕ*_*i*_, of different species *i*. Hence, the free energy (in unit of *k*_B_*T*) of the system is given by

(1) $$\begin{array}{l} F=-\varphi_{c}\ln\left(Q_{c}/\varphi_{c}V\right)-\varphi_{g}\ln\left(Q_{g}/\varphi_{g}V\right)-1/V\\ \;\int dr[\sum_{i}w_{i}\phi_{i}+\xi \left(1-\sum_{i}\phi_{i}\right)]+1/V\\ \;\int dr[\frac{1}{2}\sum_{i\neq j}\chi_{\mathrm{ij}}N\phi_{i}\phi_{j}+\sum_{i}H_{iS}N\phi_{i}\delta_{r,r_{s}}] \end{array}$$

where *χ*_*ij*_ is the Flory-Huggins interaction parameter between species *i* and *j*, *ξ* is the Lagrange multiplier (as a pressure), *η*_*i*S_ is the interaction parameter between the species *i* and the hard surface S. r_s_ is the position of the hard surfaces. *Q*_c_ = ∫dr*q*_c_(r, 1) is the partition function of a single copolymer chain in the effective external fields *w*_A_, *w*_B_, and *w*_C_, and *Q*_g_ = ∫dr*q*_g_(r, 1) is the partition function of a grafted polymer chain in the external field *w*_g_. The fundamental quantity to be calculated in mean-field studies is the polymer segment probability distribution function, *q*(r, *s*), representing the probability of finding segment *s* at position r. It satisfies a modified diffusion equation using a flexible Gaussian chain model

(2) $$\frac{\partial }{\partial s}q\left(r,s\right)=\frac{Na^{2}}{6}\nabla^{2}q\left(r,s\right)-w\left(r\right)q\left(r,s\right)$$

where *w*(r) is *w*_A_(r) when 0 < *s* < *f*_A_, *w*_B_(r) when *f*_A_ < *s* < *f*_A_ + *f*_B_, *w*_C_(r) when *f*_A_ + *f*_B_ < *s* < 1 for ABC triblock copolymer, and *w*_g_(r) for the grafted polymer. The initial condition of Equation (2) satisfies *q*_c_(r, 0) = 1 for ABC triblock copolymer. Because the two ends of the block copolymer are different, a second distribution function $q_{c}^{+}\left(r,\;s\right)$ is needed which satisfies Equation (2) but with the right-hand side multiplied by -1 and the initial condition $q_{c}^{+}\left(r,\;1\right)=1.$ The initial condition of *q*_g_(r, *s*) for grafted polymer is *q*_g_(r, 0) = *δ*(r - r_S_), where r_S_ represents the position of the substrates, and that of $q_{g}^{+}\left(r,\;s\right)$ is $q_{g}^{+}\left(r,\;1\right)=1.$ The periodic boundary condition is used for $\begin{array}{ccc} q_{c}\left(r,\;s\right), & q_{c}^{+}\left(r,\;s\right), & q_{g}\left(r,\;s\right) \end{array},$ and $q_{g}^{+}\left(r,\;s\right)$ along *x*- and *y*-directions when *z*∈ [0,*L*_*z*_]. $\begin{array}{ccc} q_{c}\left(r,\;s\right), & q_{c}^{+}\left(r,\;s\right), & q_{g}\left(r,\;s\right) \end{array},$ and $q_{g}^{+}\left(r,\;s\right)$ are equal to zero when *z* ≤ 0 or *z* ≥ *L*_z_.

Minimization of the free energy with respect to density, pressure, and fields, δ*F*/δ*ϕ =* δ*F*/δ*ξ =* δ*F*/δ*w =* 0, leads to the following equations.

(3) $$w_{A}\left(r\right)=\sum_{i\neq A}\chi_{Ai}N\phi_{i}\left(r\right)+\xi \left(r\right)+H_{\mathrm{AS}}N\delta_{r,r_{s}}$$

(4) $$w_{B}\left(r\right)=\sum_{i\neq B}\chi_{Bi}N\phi_{i}\left(r\right)+\xi \left(r\right)+H_{\mathrm{BS}}N\delta_{r,r_{s}}$$

(5) $$w_{C}\left(r\right)=\sum_{i\neq C}\chi_{Ci}N\phi_{i}\left(r\right)+\xi \left(r\right)+H_{\mathrm{CS}}N\delta_{r,r_{s}}$$

(6) $$w_{g}\left(r\right)=\sum_{i\neq g}\chi_{gi}N\phi_{i}\left(r\right)+\xi \left(r\right)+\chi_{\mathrm{gS}}N\delta_{r,r_{s}}$$

(7) $$\phi_{A}\left(r\right)+\phi_{B}\left(r\right)+\phi_{C}\left(r\right)+\phi_{g}\left(r\right)=1$$

(8) $$\phi_{A}\left(r\right)=\frac{\varphi_{c}V}{Q_{c}}\int_{0}^{f_{A}}dsq_{c}\left(r,s\right)q_{c}^{+}\left(r,s\right)$$

(9) $$\phi_{B}\left(r\right)=\frac{\varphi_{c}V}{Q_{c}}\int_{f_{A}}^{f_{A}+f_{B}}dsq_{c}\left(r,s\right)q_{c}^{+}\left(r,s\right)$$

(10) $$\phi_{C}\left(r\right)=\frac{\varphi_{c}V}{Q_{c}}\int_{f_{A}+f_{B}}^{1}dsq_{c}\left(r,s\right)q_{c}^{+}\left(r,s\right)$$

(11) $$\phi_{g}\left(r\right)=\frac{\varphi_{g}V}{Q_{g}}\int_{\;0}^{\;1}dsq_{g}\left(r,s\right)q_{g}^{+}\left(r,s\right)$$

Equations (3) to (11) form a close set of self-consistent equations, which are numerically implemented by a combinatorial screening algorithm proposed by Drolet and Fredrickson [65,66]. The algori3thm consists of randomly generating the initial values of the fields *w*_*i*_(r). Then, the diffusion equations are then integrated to obtain *q* and *q*^+^, for 0 < *s <* 1. The right-hand sides of Equations (8) to (11) are evaluated to obtain new values for the volume fractions of blocks A, B, and C, and grafted polymers. Moreover, the brief introduction of SCFT method can be found in some textbook, such as *Statistical Physics of Polymers: an Introduction*[67].

The polymerization of ABC triblock copolymer is *N* = 60 and that of the grafted chains is the same with the copolymers, i.e*.*, *P* = *N* = 60. The grafting density of the grafted chains is set as *σ* = 0.15 and 0.2 to insure that the polymer brush is in the dry brush regime (*σN*^1/2^ > 1) [68]. The interaction parameters *H*_*i*S_ (*i* = A, B, C) between the surfaces and the blocks are set to zero (the effect of the surface on the thin film is weakened because the surface is coated by polymer brushes), that means that the substrates are neutral. We only address the thin films of ABC triblock copolymer confined between densely polymer-grafted surfaces, and the grafted polymers are assumed to be identical with the middle block B. We continuously vary the compositions to search the morphology of the ABC block copolymer thin film. The simulations are performed on a 3D cubic box *L*_*x*_ × *L*_*y*_ × *L*_*z*_. The two parallel hard surfaces are presented as planes at *z* = 0 and *L*_*z*_ + *a*, and the film thickness is set to *L*_*z*_ *=* 40*a*, which is appropriate for thin film with the effective thickness of several *R*_g_. *L*_*x*_ and *L*_*y*_ along *xy*-plane can be varied between 40 to 45*a* to avoid the size effect and obtain the stable and perfect morphology. It should be noted that the resulting microphases largely depend on the initial conditions. Therefore, all the simulations are repeated many times using different random states to guarantee the structure is not occasionally observed. In this work, three cases are considered: (1) identical interactions between three different components, *χ*_AB_*N* = *χ*_BC_*N* = *χ*_AC_*N* = 35, which are widely studied in many theoretical works; (2) frustrated condition *χ*_AB_*N* = *χ*_BC_*N* = 35 and *χ*_AC_*N* = 13; and (3) non-frustrated condition, *χ*_AB_*N* = *χ*_BC_*N* = 13 and *χ*_AC_*N* = 35 based on the work of Jung [69] and Tyler [1]. Furthermore, the effect of the brush density is also included in the case of *χ*_AB_*N* = *χ*_BC_*N* = *χ*_AC_*N* = 35, which is actually equivalent to changing the effective film thickness.

## Results and discussion

**Figure 1:**
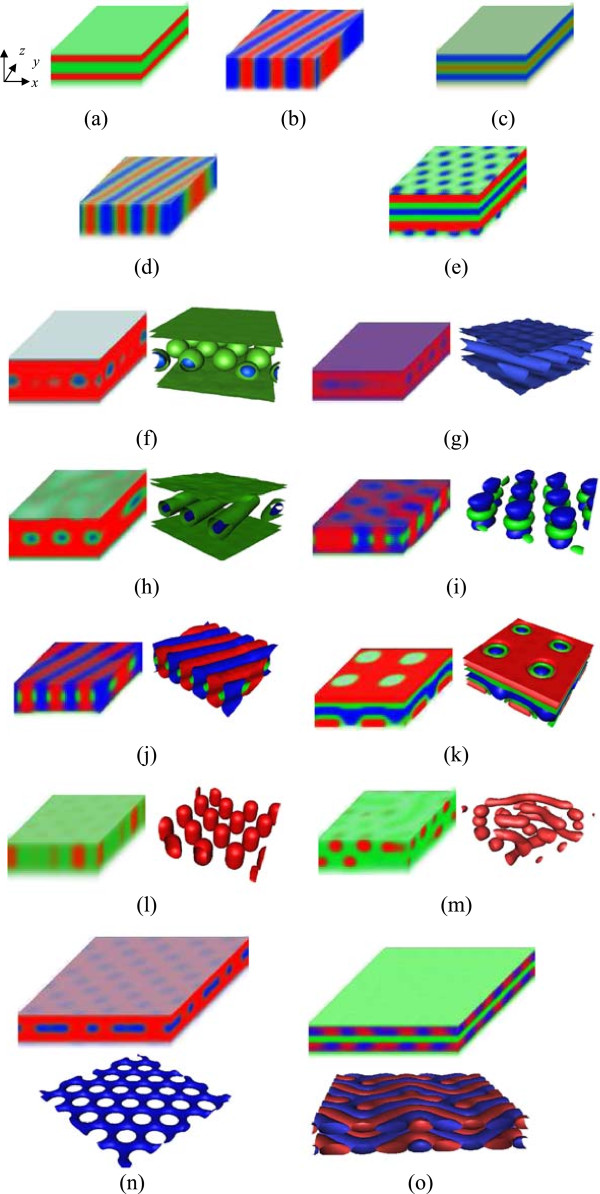
**Morphologies of the ABC block copolymer thin film with *L***_***z***_ ***=*** **40*a*.** The microphase patterns, displayed in the form of density, are the red, green, and blue, assigned to A, B, and C, respectively. For some morphologies, the 3D isosurface graphs are also given for a clear view beside the morphologies. The red, green, and blue colors in isosurface graphs are assigned to blocks A, B, and C for a good correspondence, respectively. **(a)** Two-color parallel lamellar phase (LAM_2_^*ll*^), **(b)** two-color perpendicular lamellar phase (LAM_2_^⊥^), **(c)** three-color parallel lamellar phase (LAM_3_^*ll*^), **(d)** three-color perpendicular lamellar phase (LAM_3_^⊥^), **(e)** parallel lamellar phase with hexagonally packed pores at surfaces (LAM_3_^*ll*^-HFs), **(f)** core-shell hexagonally packed spherical phase (CSHS), **(g)** two-color parallel cylindrical phase (C_2_^*ll*^), **(h)** core-shell parallel cylindrical phase (CSC_3_^*ll*^), **(i)** perpendicular hexagonally packed cylindrical phase with rings at the interface (C_2_^⊥^-RI), **(j)** perpendicular lamellar phase with cylinders at the interface (LAM^⊥^-CI), **(k)** parallel lamellar phase with tetragonal pores (LAM_3_^*ll*^-TF), **(l)** perpendicular hexagonally packed cylindrical phase (C_2_^⊥^), **(m)** sphere-cylinder transition phase (S-C), **(n)** hexagonal pores (HF), and **(o)** irregular lamellar phase (LAM_i_). Morphologies in **(n)** and **(o)** are enlarged by two times along *x*- and *y*-directions.

Figure 1 presents the morphologies of the ABC triblock copolymer thin film by varying the compositions of the block copolymer. The microphase patterns, displayed in the form of density, are the red, green, and blue, assigned to blocks A, B, and C, respectively. The final color plotted at each point is the mixture of three colors, in which the concentration of each color is proportional to the local volume fraction of an individual block. The 3D morphology can only give the three faces (*xy*, *yz*, *xz*) of the ABC triblock copolymer thin film. For some morphologies, the 3D isosurface graphs are also given for a clear view. The red, green, and blue colors in isosurface graphs are assigned to blocks A, B, and C for a good correspondence, respectively. In these 3D isosurface graphs, some only give one or two components. Here, we do not show the morphologies of the polymer brushes in order to clearly see the morphologies of the block copolymer. There are at least 15 stable morphologies found: two-color parallel lamellar phase (LAM_2_^*ll*^), two-color perpendicular lamellar phase (LAM_2_^⊥^), three-color parallel lamellar phase (LAM_3_^*ll*^), three-color perpendicular lamellar phase (LAM_3_^⊥^), parallel lamellar phase with hexagonally packed pores at surfaces (LAM_3_^*ll*^-HFs), two-color parallel cylindrical phase (C_2_^*ll*^), core-shell hexagonally packed spherical phase (CSHS), core-shell parallel cylindrical phase (CSC_3_^*ll*^), perpendicular lamellar phase with cylinders at the interface (LAM^⊥^-CI), perpendicular hexagonally packed cylinders phase with rings at the interface (C_2_^⊥^-RI), parallel lamellar phase with tetragonal pores (LAM_3_^*ll*^-TF), perpendicular hexagonally packed cylindrical phase (C_2_^⊥^), sphere-cylinder transition phase (S-C), hexagonal pores (HF), and irregular lamellar phase (LAM_i_). In these morphologies, there are some interesting structures, such as LAM_3_^*ll*^-HFs, LAM^⊥^-CI, LAM_3_^*ll*^-TF, and HF. HF phase is also experimentally observed [60], which is very useful; for example, the perforated lamella can serve as a lithographic mask. There are two irregular phases, sphere-cylinder transition phase (S-C) and irregular lamellar phase (LAM_i_). Due to the composition and the surface interaction competition, it is difficult to form the regular and stable phase. In fact, the parallel lamellar phases have three different arrangement styles near the brush. Because the brushes are identical to the middle block B, the block B should be near the brushes. But it is not always the case due to entropic effect. So, the blocks A, B, or C can be adjacent to the brushes. So in the following phase diagrams, we discern the three different arrangement styles of the parallel lamellar phases. When the block B is major in the block copolymer, the parallel lamellar phase with block B adjacent to brush layer is stable. When the block B is minor, the parallel lamellar phase with block A or B adjacent to brush layer is stable.

(1) Identical interaction parameter *χ*_AB_*N* = *χ*_BC_*N* = *χ*_AC_*N* = 35.

a. Influence of the composition

**Figure 2:**
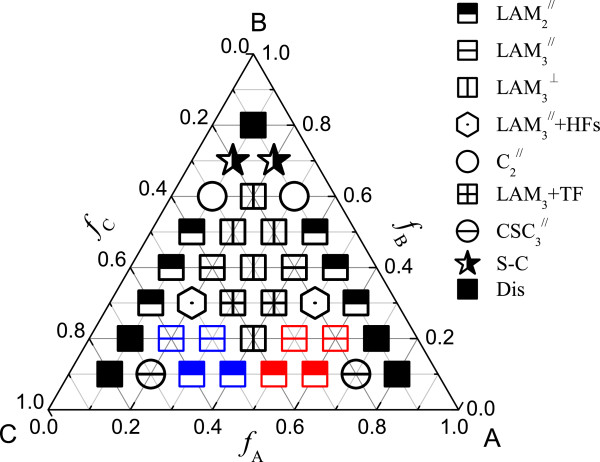
**Phase diagram of ABC triblock copolymer with *χ***_**AB**_***N*** **=** ***χ***_**BC**_***N*** **=** ***χ***_**AC**_***N*** **= 35 at grafting density*σ*** **= 0.20.** Dis represents the disordered phase. The red, blue, or black icons showing the parallel lamellar phases discern the different arrangement styles of the block copolymer with block A, block C, or block B adjacent to the brush layers, respectively.

In this part, we consider the case of *χ*_AB_*N* = *χ*_BC_*N* = *χ*_AC_*N* = 35. Figure 2 gives the phase diagram of the ABC triblock copolymer when the brush density *σ* is 0.2. There are nine phases in the diagram. Due to the confinement of the ABC triblock copolymer and the tailoring effect of polymer brushes, the diagram is largely different from that in the bulk [33].

The disordered phase (Dis) exists at the three corners of the phase diagram. When the volume fractions of the three blocks are comparable, the three-color lamellar phase forms, which is similar with that in the bulk [33]. When one of the blocks is the minority, the phase behavior is similar with that of the diblock copolymer. When the middle block B is the minority, most of block B will accumulate between the A/C interface, and while the end block A or C is the minority, other block C or A will distribute in the middle block B to form one phase. There are many two-color phases near the edges of the phase diagram, and at this time, the lamellar phase is parallel to the surfaces. This shows that we can add a small functional block A or C to symmetric BC or AB diblock copolymer to obtain a lamellar phase parallel to the surfaces.

The diagram has the A-C reflectivity due to the symmetric architecture and the symmetric interaction parameters. When the volume fractions of the blocks A and C are comparable, the perpendicular lamellar phase easily forms except *f*_B_ ≤ 0.1 and *f*_B_ ≥ 0.7 and the compositions *f*_A_ = 0.3, *f*_B_ = 0.3, *f*_C_ = 0.4, and *f*_A_ = 0.4, *f*_B_ = 0.3, *f*_C_ = 0.3.

b. Influence of the grafting density

**Figure 3:**
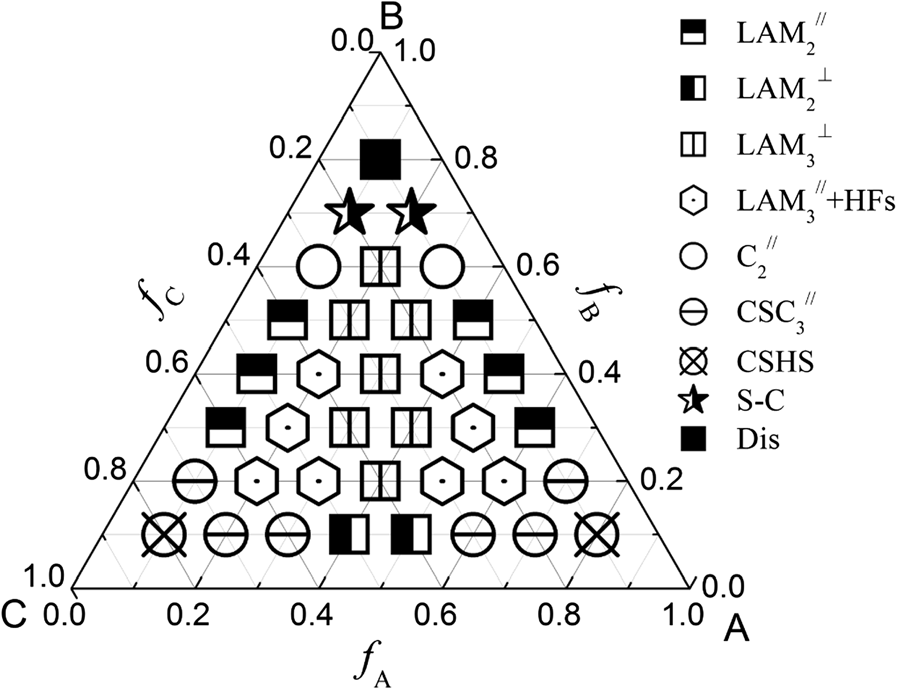
**Phase diagram of ABC triblock copolymer with *χ***_**AB**_***N*** **=** ***χ***_**BC**_***N*** **=** ***χ***_**AC**_***N*** **= 35 at grafting density*σ*** **= 0.15.** Dis represents the disordered phase.

We also consider the grafting density *σ =* 0.15 when *χ*_AB_*N* = *χ*_BC_*N* = *χ*_AC_*N* = 35. The grafting density decreases a little, which shows that the effective film thickness increases. The phase diagram is shown in Figure 3. From the figure, we can see that the lamellar phase region contracts and some new phases emerge, such as two-color perpendicular lamellar phase (LAM_2_^⊥^) and core-shell hexagonally packed spherical phase (CSHS). Due to the decrease of the grafting density, the influence of the brush will weaken. Similar with the case of *σ* = 0.20, the core-shell structures occur near the corners A and C. CSHS phase forms at *f*_A_ = 0.10, *f*_B_ = 0.10, *f*_C_ = 0.80; *f*_A_ = 0.80, *f*_B_ = 0.10, *f*_C_ = 0.10. The core-shell cylindrical phase occurs near the phase CSHS. In these cases, the block A (or C) forms the majority, the block C (or A) forms the ‘core,’ and the middle block B is around the block C (or A) forming the ‘shell’ of the core.

Comparing the phase diagram with that in the bulk [33], the direction of the lamellar phase can be tailored by changing the grafting density when the middle blocks are the minority and the ABC triblock copolymer is symmetric, i.e. *f*_A_ = *f*_C_. The parallel lamellar phase with hexagonally packed pores at surfaces (LAM_3_^*ll*^-HFs) can easily form at some compositions.

In general, the block copolymer experiences the film confinement under this condition. Moreover, the block copolymer experiences the brush polymer tailoring, especially at the interface between the block copolymer and the polymer brush. Therefore, some new phases form, and the phase diagram is more complicated. Even for the lamellar phase, there are two styles: the perpendicular and parallel ones. The perpendicular lamellar phase always occurs when the volume fractions of the three components are comparable. The parallel lamellar phase forms at the middle edge of the phase diagram in most cases. From the above two phase diagrams, we can see that the hexagonally packed pores at the interface between the block copolymers and the polymer brush-coated surfaces occur. It is very useful in designing thin films with functional dots.

(2)  Frustrated case *χ*_AB_*N* = *χ*_BC_*N* = 35, *χ*_AC_*N* = 13

**Figure 4:**
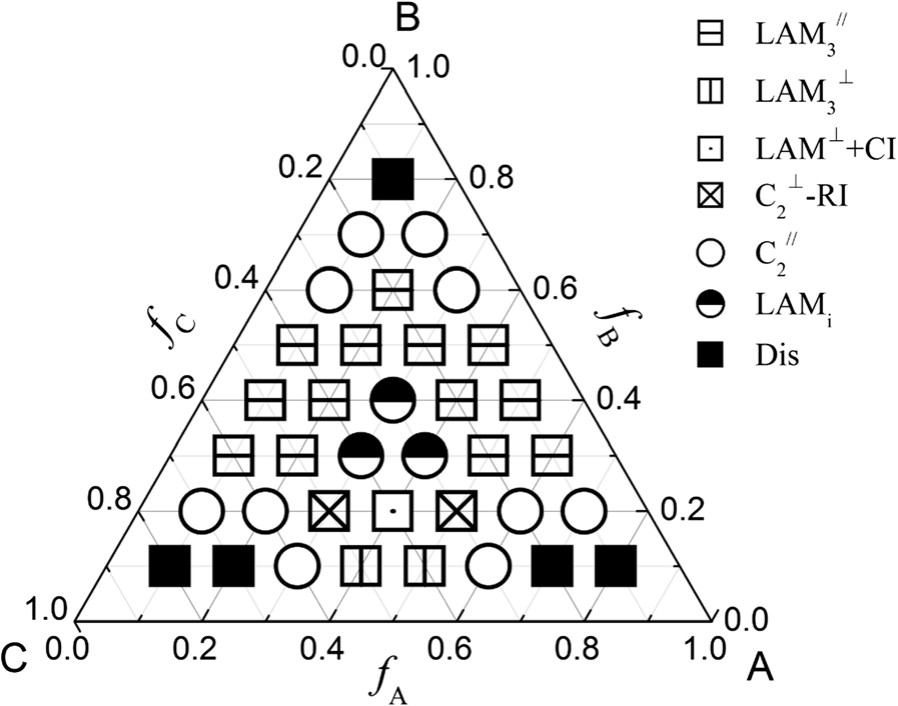
**Phase diagram of ABC triblock copolymer with *χ***_**AB**_***N*** **=** ***χ***_**BC**_***N*** **= 35 and*χ***_**AC**_***N*** **= 13 at grafting density*σ*** **= 0.2.** Dis represents the disordered phase.

It is energetically unfavorable when *χ*_AC_*N* < < *χ*_AB_*N* ≈ *χ*_BC_*N*; that is to say, the repulsive interaction between the two ends is the smallest in the three interaction parameters. Thus, the block B has to be limited in spheres, rings, or cylinders to increase the contacting interface between the blocks A and C. Some phases are also observed in the frustrated ABC triblock copolymer in bulk [70], such as C_2_^⊥^-RI and LAM^⊥^-CI (which correspond to C + HEL and L + C(II) in [56]). The phase diagram is shown in Figure 4 for *χ*_AB_*N* = *χ*_BC_*N* = 35 and *χ*_AC_*N* = 13.

Due to the energetic confinement, the two-color lamellar phase is easy to form. When the middle block B is the minority, the phases are complex. The block B will accumulate near the interface between the blocks A and C, which can be comparable with that in the bulk in the frustrated case [33,70]. For the symmetric ABC triblock copolymer, i.e., *f*_A_ = *f*_C_, with the increase of the volume fraction of the middle block B, the phase will change from the perpendicular lamellar phase to perpendicular lamellar phase with cylinders at the interface to irregular lamellar phase to three-color parallel lamellar phase. This shows that the direction of the lamellar phase can be tailored. The irregular lamellar phase (three points *f*_A_ = 0.3, *f*_B_ = 0.3, *f*_C_ = 0.4; *f*_A_ = 0.4, *f*_B_ = 0.3, *f*_C_ = 0.3; *f*_A_ = 0.3, *f*_B_ = 0.4, *f*_C_ = 0.3) forms because of two reasons: one is the three blocks with almost equal volume fraction, and the middle block B will stay near to the polymer-coated (same with block B) substrates, so there is not enough block B to form the perfect lamellar phase. The other reason is *χ*_AC_*N* < < *χ*_AB_*N* ≈ *χ*_BC_*N*, then the copolymer chain will overcome the elastic energy to form the A/C interface. Therefore, the phase is not perfect because of the composition competition and the energy competition. And the most important is that perpendicular hexagonally packed cylindrical phase with rings at the interface (C_2_^⊥^-RI) and perpendicular lamellar phase with cylinders at the interface (LAM^⊥^-CI) occur in this frustrated case, see Figure 1i,j. In fact, these two phases are obtained in the frustrated ABC triblock copolymer with interaction parameters *χ*_AB_*N* = *χ*_BC_*N* = 35 and *χ*_AC_*N* = 15 in bulk [70].

(3)  Non-frustrated case (*χ*_AB_*N* = *χ*_BC_*N* = 13, *χ*_AC_*N* = 35)

**Figure 5:**
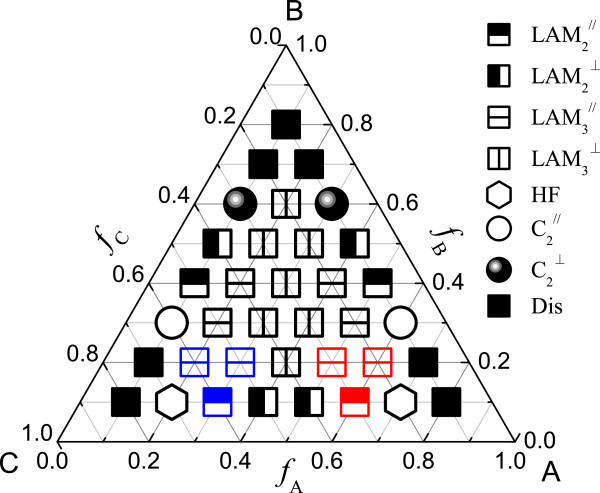
**Phase diagram of ABC triblock copolymer with *χ***_**AB**_***N*** **=** ***χ***_**BC**_***N*** **= 13 and *χ***_**AC**_***N*** **= 35 at grafting density*σ*** **= 0.2.** Dis represents the disordered phase. The red, blue, or black icons showing the parallel lamellar phases discern the different arrangement styles of the block copolymer with block **A**, block **C**, or block **B** adjacent to the brush layers, respectively.

It is an energetically favorable case when the repulsive interaction between the end blocks A and C is larger than that for blocks A and B or blocks B and C. Here, we consider the case of *χ*_AB_*N* = *χ*_BC_*N* = 13 and *χ*_AC_*N =* 35, which is used when considering the non-frustrated case for ABC block copolymer [1]. The phase diagram of ABC triblock copolymer thin film for *χ*_AB_*N* = *χ*_BC_*N* = 13 and *χ*_AC_*N =* 35 is shown in Figure 5. Eight phases are found in this case. Due to the relative weak interaction between the blocks A and B and between the blocks B and C, the disordered phase occurs at the corners of the three blocks. The lamellar phase region is very large. The three-color lamellar phase forms when the volume fractions of the three components are comparable. The two-color lamellar phase is stable in the middle of the three edges in the phase diagram. When the volume fractions of the blocks A and C are equal, the perpendicular lamellar phase easily forms between *f*_B_ = 0.1 and 0.6.

(4)  Comparison with ABC triblock copolymer thin film without polymer brush-coated substrates

**Figure 6:**
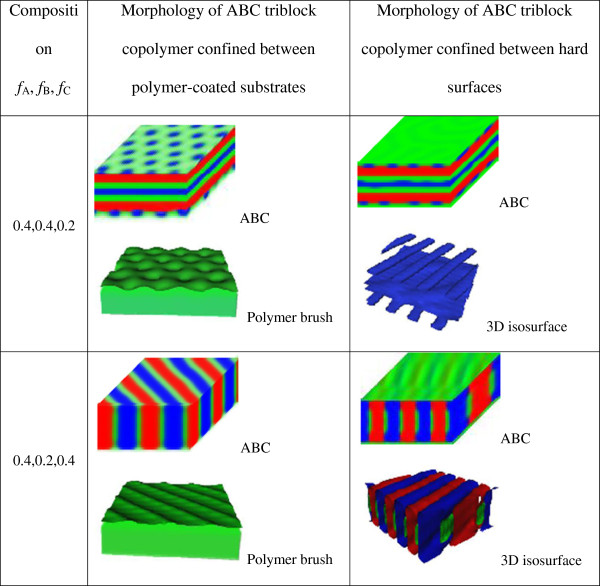
**Comparison of the morphology of ABC triblock copolymer confined between hard surfaces and polymer brush-coated substrates.** The microphase patterns, displayed in the form of density, are the red, green, and blue, assigned to A, B, and C, respectively. The 3D isosurface graphs are also given for a clear view for the ABC triblock copolymer confined between the hard surfaces. The red, green, and blue colors in isosurface graphs are assigned to the blocks A, B, and C for a good correspondence, respectively. For the ABC triblock copolymer confined between polymer brush-coated substrates, the 3D isosurface of the grafted polymer on the lower substrate is also shown below the morphology due to the symmetry of the polymer brush. For the ABC triblock copolymer confined between hard surfaces, the 3D isosurface is also shown below the morphology.

In this part, we give two cases for comparison between the ABC triblock copolymer thin film with and without polymer brush-coated substrates (*σ* = 0.15) at *χ*_AB_*N* = *χ*_BC_*N* = *χ*_AC_*N* = 35. In order to simulate the similar interface environment with the ABC triblock copolymer thin film between polymer brush-coated substrates, we set the interaction parameters *η*_AS_*N* = *η*_CS_*N* = 35 and *η*_BS_*N* = 0 for the ABC triblock copolymer thin film between hard surfaces, which means the substrate is good for the middle block B. In principle, the effective film thickness for the ABC triblock copolymer thin film confined between the polymer brush-coated substrates is like *L*_*z*_^eff^ = *L*_*z*_ - 2*aσP* for *σP*^1/2^ > 1 (where 2 is just for the upper and lower polymer grafted surfaces, brush height *h* = *aσP* for *σP*^1/2^ > 1 [68]). When the ABC triblock copolymer is confined between two hard surfaces (without polymer brush-coated substrates), the corresponding effective film thickness is 22*a* in this case.The morphology comparison of ABC triblock copolymer confined between polymer-coated substrates and hard surfaces is listed in Figure 6. The first column is the composition of ABC triblock copolymer. The second column is the morphologies of the ABC triblock copolymer confined between the polymer brush-coated surfaces and the morphologies of the polymer brush. The third column is the morphologies of ABC triblock copolymer confined between hard surfaces (without polymer brush-coated) and the 3D isosurface for a clear view. The microphase patterns, displayed in the form of density, are the red, green, and blue, assigned to A, B, and C, respectively. Similarly, the red, green, and blue colors in 3D isosurface graphs are assigned to blocks A, B, and C for a good correspondence, respectively. For the ABC triblock copolymer confined between polymer brush-coated substrates, the morphology of the grafted polymer on the lower substrate (polymer brush) is also shown below the morphology of ABC triblock copolymer. We only give the morphology of the grafted polymer on the lower substrate (polymer brush) due to the symmetry of the polymer brush (the two polymer brush-coated surfaces are identical). For the ABC triblock copolymer confined between the hard surfaces, the 3D isosurface is also shown below the morphology.

When *f*_A_ = 0.4, *f*_B_ = 0.4, and *f*_C_ = 0.2 at *σ* = 0.15, the phase LAM_3_^*ll*^-HFs is stable, while the stable phase for the thin film confined between hard surfaces is three-color lamellae with parallel cylinders at the interfaces. When *f*_A_ = 0.4, *f*_B_ = 0.2, and *f*_C_ = 0.4 at *σ* = 0.15, the perpendicular lamellar phase LAM_3_^⊥^ is stable, while the perpendicular lamellar phase with cylinder at the interfaces is stable without the coated polymer brush at the surfaces. From the morphology of the polymer brush, we can see that there is some ordered pattern at the interface between the thin film and the polymer brush. So, we think the coated polymers on the substrates have penetrated into the ABC triblock copolymer thin film, and the interaction between them contributes to morphology formation of the thin film. For the case of *f*_A_ = 0.4, *f*_B_ = 0.2, and *f*_C_ = 0.4, the perpendicular lamellar phase with cylinders at the interfaces is stable without the coated polymer brush at the surfaces. But when it is confined between the polymer brush-coated substrates, the polymer brush will penetrate into the block copolymer thin film and form one phase with the middle block B, so the perpendicular lamellar phase occurs.

**Figure 7:**
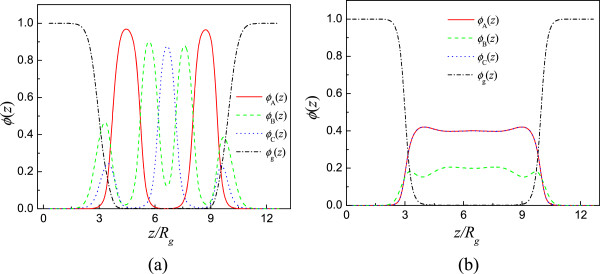
**Density distribution of the different components along *z*-direction with*χ***_**AB**_***N*** **=** ***χ***_**BC**_***N*** **=** ***χ***_**AC**_***N*** **= 35,*σ*** **= 0.15. (a)***f*_A_ = 0.4, *f*_B_ = 0.4, *f*_C_ = 0.2; **(b)***f*_A_ = 0.4, *f*_B_ = 0.2, *f*_C_ = 0.4.

The density profile of the block copolymer along *z*-direction can be obtained by $\phi_{i}\left(z\right)=\sum_{x,y}\phi_{i}\left(x,y,z\right)/\left(L_{x}L_{y}/a^{2}\right)$ (*i* belongs to blocks A, B, and C and grafting polymer g). Figure 7 gives the density profiles of the blocks A(solid), B(dash), and C(dot) and the grafting polymer(dash dot) for the cases (a) *f*_A_ = 0.4, *f*_B_ = 0.4, and *f*_C_ = 0.2 and (b) *f*_A_ = 0.4, *f*_B_ = 0.2, *f*_C_ = 0.4. The polymer brush and the middle block B have interpenetration. So, the interfacial morphology is different from the block copolymer confined between hard surfaces. We can see the lamellar distribution parallel to the substrates for *f*_A_ = 0.4, *f*_B_ = 0.4, *f*_C_ = 0.2, so there are peaks along *z*-direction which correspond to the domain centers of the blocks. The perpendicular lamellar phase forms for *f*_A_ = 0.4, *f*_B_ = 0.2, *f*_C_ = 0.4, and the uniform distribution exists in the middle of the film. The curves for the blocks A and C are overlapped due to the same composition and the symmetric interaction parameters between different blocks. These density profiles clearly show the coated polymer brush attributes to the morphology formation of the block copolymer. We can use the polymer brush to tailor the morphology of the block copolymer thin film.

## Conclusions

The morphology and the phase diagrams of ABC triblock copolymer thin film confined between polymer brush-coated surfaces are investigated by the real-space self-consistent field theory in three dimensions. The coated polymer brush is identical with the middle block B. By continuously changing the composition of the block copolymer, the phase diagrams are constructed for three cases with the fixed film thickness *L*_*z*_ = 40*a* and the grafting density *σ* = 0.20: (1) identical interactions between three different components, *χ*_AB_*N* = *χ*_BC_*N* = *χ*_AC_*N* = 35; (2) frustrated condition *χ*_AB_*N* = *χ*_BC_*N* = 35 and *χ*_AC_*N* = 13; and (3) non-frustrated condition, *χ*_AB_*N* = *χ*_BC_*N* = 13 and *χ*_AC_*N* = 35. Furthermore, the brush density *σ* = 0.15 is also included in the case of *χ*_*AB*_*N* = *χ*_BC_*N* = *χ*_AC_*N* = 35. Fifteen stable morphologies are obtained: LAM_2_^*ll*^, LAM_2_^⊥^, LAM_3_^*ll*^, LAM_3_^⊥^, LAM_3_^*ll*^-HFs, C_2_^*ll*^, CSHS, CSC_3_^*ll*^, LAM^⊥^-CI, C_2_^⊥^-RI, LAM_3_^*ll*^-TF, C_2_^⊥^, S-C, HF, and LAM_i_. The morphology of the block copolymer thin film largely depends on the compositions and the surface interaction besides the film thickness. The complex morphology can be obtained at the energetically unfavorable condition, such as the cases for *χ*_AB_*N* = *χ*_BC_*N* = *χ*_AC_*N* = 35 and *χ*_AB_*N* = *χ*_BC_*N* = 35 and *χ*_AC_*N* = 13. Although the grafted polymers are identical to the middle block B, the perpendicular lamellar phase is not always the stable one. The perpendicular or parallel lamellar phases can be obtained by varying the composition (besides changing the film thickness) and the interactions between different blocks. When one of the end block A or C is minority, the two-color parallel lamellar phase easily forms, while the perpendicular lamellar phase is stable when the block copolymer is symmetric, i.e., *f*_A_ = *f*_C_. Even the direction of the cylinders can also be tuned for the non-frustrated case, where the direction of the cylinder can be tailored by the composition of the block. The parallel cylindrical phase forms if the end block A or C is the majority (*f*_A_ or *f*_C_ = 0.6), and the perpendicular cylindrical phase forms if the middle block B is the majority (*f*_B_ = 0.6) for the non-frustrated case. There are some interesting phases, such as hexagonally packed pores at surfaces (LAM_3_^*ll*^ + HFs) and perpendicular hexagonally packed cylindrical phase with rings at the interface (C_2_^⊥^-RI). Compared with the case of the ABC triblock copolymer thin film without polymer brush-coated substrate, the morphologies of ABC triblock copolymer thin film confined between polymer brush-coated substrates show some preferences and are easily controllable. The perpendicular lamellar phase is stable when the block copolymer is symmetric, i.e., *f*_A_ = *f*_C_ unlike the case of thin film without polymer brush-coated substrate, the direction will change at different film thickness. Although the grafted polymers are identical to the middle block B, the perpendicular lamellar phase is not always the stable one. The perpendicular or parallel lamellar phases can be obtained by varying the composition and the interactions between different blocks. Even the direction of the cylinders can also be tuned for the non-frustrated case. Our simulation results give an overview of ABC triblock copolymer thin film confined between the polymer brush-coated surfaces and are very useful in designing the complex morphology of ABC triblock copolymer thin film; for example, we can obtain the LAM_3_^*ll*^-HFs, which is potentially useful in designing the functional dots near the surfaces.

## Competing interests

The authors declare that they have no competing interests.

## Authors’ contributions

ZBJ, CX, and YDQ carried out the simulations. ZBJ performed the data analysis and drafted the manuscript and participated in its design. XLW, DSZ, and GX participated in the design of the study and conceived of the study. All authors read and approved the final manuscript.
